# Development and preliminary assessment of a CRISPR–Cas12a-based multiplex detection of *Mycobacterium tuberculosis* complex

**DOI:** 10.3389/fbioe.2023.1233353

**Published:** 2023-08-25

**Authors:** Jing Xiao, Jieqiong Li, Shuting Quan, Yacui Wang, Guanglu Jiang, Yi Wang, Hairong Huang, Weiwei Jiao, Adong Shen

**Affiliations:** ^1^ Laboratory of Respiratory Diseases, Beijing Key Laboratory of Pediatric Respiratory Infection Diseases, Key Laboratory of Major Diseases in Children, National Center for Children’s Health, National Clinical Research Center for Respiratory Diseases, National Key Discipline of Pediatrics (Capital Medical University), Beijing Pediatric Research Institute, Ministry of Education, Beijing Children’s Hospital, Capital Medical University, Beijing, China; ^2^ Medical Research Center, Beijing Institute of Respiratory Medicine, Beijing Chao-Yang Hospital, Capital Medical University, Beijing, China; ^3^ National Tuberculosis Clinical Laboratory, Beijing Key Laboratory for Drug Resistance Tuberculosis Research, Beijing Tuberculosis and Thoracic Tumor Research Institute, Beijing Chest Hospital, Capital Medical University, Beijing, China; ^4^ Experimental Research Center, Capital Institute of Pediatrics, Beijing, China

**Keywords:** *Mycobacterium tuberculosis* complex, CRISPR-Cas12a, multiplex detection, fluorescent biosensor, lateral flow biosensor, point-of-care testing

## Abstract

Since the onset of the COVID-19 pandemic in 2020, global efforts towards tuberculosis (TB) control have encountered unprecedented challenges. There is an urgent demand for efficient and cost-effective diagnostic technologies for TB. Recent advancements in CRISPR–Cas technologies have improved our capacity to detect pathogens. The present study established a CRISPR–Cas12a-based multiplex detection (designated as MCMD) that simultaneously targets two conserved insertion sequences (IS*6110* and IS*1081*) to detect *Mycobacterium tuberculosis* complex (MTBC). The MCMD integrated a graphene oxide-assisted multiplex recombinase polymerase amplification (RPA) assay with a Cas12a-based trans-cleavage assay identified with fluorescent or lateral flow biosensor (LFB). The process can be performed at a constant temperature of around 37°C and completed within 1 h. The limit of detection (LoD) was 4 copies μL^−1^, and no cross-reaction was observed with non-MTBC bacteria strains. This MCMD showed 74.8% sensitivity and 100% specificity in clinical samples from 107 patients with pulmonary TB and 40 non-TB patients compared to Xpert MTB/RIF assay (63.6%, 100%). In this study, we have developed a straightforward, rapid, highly sensitive, specific, and cost-effective assay for the multiplex detection of MTBC. Our assay showed superior diagnostic performance when compared to the widely used Xpert assay. The novel approach employed in this study makes a substantial contribution to the detection of strains with low or no copies of IS6110 and facilitates point-of-care (POC) testing for MTBC in resource-limited countries.

## 1 Introduction

Before the outbreak of the coronavirus disease 2019 (COVID-19) pandemic in December 2019, tuberculosis (TB) caused by the *Mycobacterium tuberculosis* complex (MTBC) was the leading cause of death from a single infectious agent worldwide, surpassing the human immunodeficiency virus (HIV) infection (World-Health-Organization, 2020). In 2019, an estimated 10 million people developed TB worldwide, of which 29.0% went undiagnosed and unreported due to the lack of rapid screening and accurate diagnostic techniques (World-Health-Organization, 2020). However, as a result of the COVID-19 pandemic, the proportion of undiagnosed TB among the estimated incident TB worldwide increased to 42.6% (2020) and 39.6% (2021) (World-Health-Organization, 2021; World-Health-Organization, 2022). In China, this proportion increased from 12.6% in 2019 to 25.8% in 2020 and 25.0% in 2021 (World-Health-Organization, 2020; World-Health-Organization, 2021; World-Health-Organization, 2022).

The TB diagnostic tests currently available have certain limitations (MacLean et al., 2020). For example, the bacilli culture, known as the gold standard for laboratory diagnosis of TB, is laborious and time-consuming with moderate accuracy. Immunological tests such as the interferon-gamma release assay (IGRA) cannot differentiate active TB from latent TB infection (LTBI) (Walzl et al., 2018). The PCR-based Xpert MTB/RIF assay (hereinafter referred to as “Xpert”), recommended by the WHO in 2011 (World-Health-Organization, 2011), shows moderate efficacy in diagnosing paucibacillary TB, such as smear-negative pulmonary TB, extra-pulmonary TB, and pediatric TB (Kay et al., 2020; Kohli et al., 2021). Xpert MTB/RIF Ultra, the next-generation Xpert, has been shown to have an improved sensitivity. However, the high costs of using and maintaining Xpert MTB/RIF Ultra hinder their widespread applications in TB diagnostics (Kay et al., 2020; Kohli et al., 2021). Therefore, there is an urgent need to develop sensitive, efficient, and cost-effective diagnostic technologies for TB to effectively control and prevent the spread of the disease.

The clustered regularly interspaced short palindromic repeats (CRISPR)-Cas (CRISPR associated) proteins system, derived from the prokaryotic adaptive immune system, consists of a Cas endonuclease (e.g., Cas9, Cas12a, Cas12b, Cas13a, Cas13b, and Cas14) and a genetically engineered guide RNA (gRNA). It has been observed that Cas and gRNA can form an effective ribonucleoprotein (RNP) complex to degrade foreign nucleic acids complementary to the gRNA sequence. CRISPR–Cas systems have been used extensively in genome editing, gene regulation, and molecular diagnostics. Several Cas endonucleases (i.e., Cas12a, Cas12b, Cas13a, and Cas14) can trans-cleave non-target single-stranded nucleic acids (including ssDNA and ssRNA) after cleaving target nucleic acids, which is termed as collateral cleavage activities. Based on such property, several CRISPR diagnostic platforms, such as CRISPR–Cas13a-based SHERLOCK (Specific High-Sensitivity Enzymatic Reporter UnLOCKing) (Gootenberg et al., 2017), CRISPR–Cas12a-based DETECTR (DNA Endonuclease Targeted CRISPR Tans Reporter) (Chen et al., 2018), HOLMES (One-Hour-Low-cost Multipurpose highly Efficient System) (Li et al., 2018), CRISPR–Cas14-based DETECTR (Harrington et al., 2018), and CRISPR–Cas12b-based HOLMESv2 (Li et al., 2019), have been developed and used for the detection of various pathogens. The CRISPR diagnostic platforms can achieve attomolar sensitivity for detecting target nucleic acids with single-base resolution.

The present study first combined an improved isothermal amplification technique (Graphene oxide-assisted multiplex recombinase polymerase amplification assay, GO-assisted multiplex RPA assay) that simultaneously targets two conserved insertion sequences (IS*6110* and IS*1081*) with a CRISPR–Cas12a-based trans-cleavage assay for a simple, rapid, sensitive, and specific diagnosis of MTBC. The application of these techniques was subsequently validated using clinical samples. This novel and cost-effective detection technique, MTBC CRISPR–Cas12a Multiplex Detection (MCMD), can detect MTBC isolates at point-of-care (POC) in resource-constrained countries or strains with low/no copy numbers of IS*6110*.

## 2 Materials and methods

### 2.1 Reagents and instruments

The QIAamp DNA Mini Kit and glass bead-based kit used for DNA extraction were purchased from Qiagen (Hilden, Germany) and CapitalBio Technology Co., Ltd. (Beijing, China). The TwistAmp Basic Kit used for isothermal amplification was purchased from TwistDx (Cambridge, United Kingdom). Graphene oxide (2 mg mL^−1^) was purchased from Sigma Aldrich (MO, United States), and the CRISPR–Cas enzyme LbCas12a was obtained from GenScript Biotechnology Co., Ltd. (Nanjing, China). Primers, gRNAs, and ssDNA reporter molecules were synthesized by Qingke Biotechnology Co., Ltd. (Beijing, China). Lateral flow biosensors (LFB) were manufactured by HuiDeXing Biotechnology Co., Ltd. (Tianjin, China). DNA concentration was determined using the Nanodrop 2000 instrument (ThermoFisher Scientific, United Kingdom). Isothermal amplification and quantitative fluorescence PCR were performed using Eppendorf AG pro S Mastercycler (Eppendorf, Germany) and the AriaMx Real-Time PCR system (Agilent Technologies, CA, United States), respectively. The gel was imaged with the Gel Doc XR + Imaging System (Bio-Rad, CA, United States).

### 2.2 Primers, gRNAs and ssDNAs design

**FIGURE 1:**
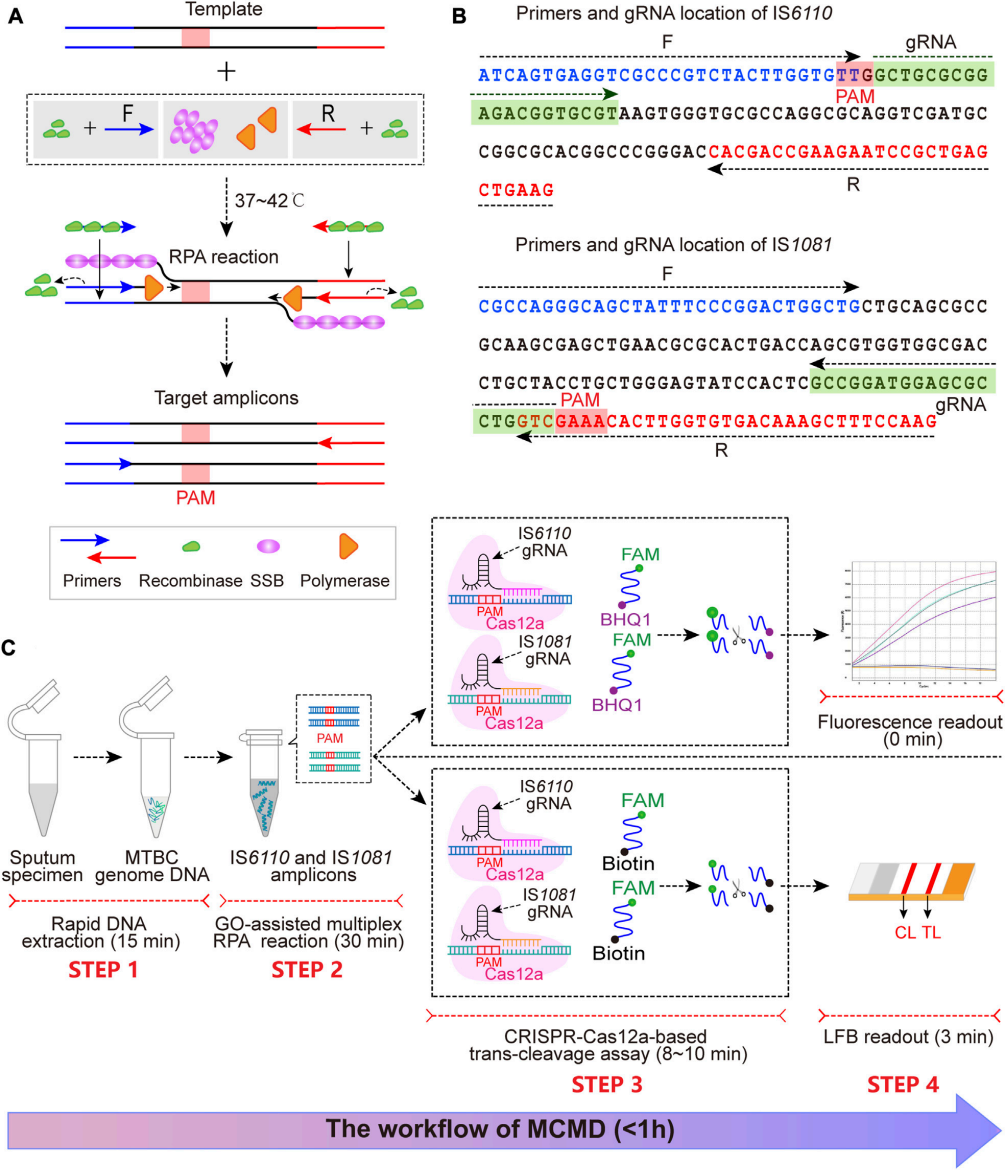
The schematic illustration of MCMD workflow. **(A)** The schematic diagram of RPA. At 37°C–42°C, recombinase (green)-primer (blue/red arrow) complexes scan the double-stranded DNA for homologous sequences (blue/red line segments), promoting the initiation of a strand exchange event at the cognate sites. The displaced template strand binds to SSB (magenta) to prevent the ejection of the primer by branch migration. The recombinase disassembles from the primer, which leaves the 3′-end of the oligonucleotide accessible to a DNA polymerase (orange), and this is followed by primer extension. Repetition of this procedure results in exponential amplification of DNA. The PAM sequence is presented in the red box. **(B)** The primer and gRNA design of MCMD. Nucleotide sequences of the amplification products from IS*6110* and IS*1081* are shown. Nucleotide sequences in blue and red are the binding positions of forward (F) and reverse (R) primers, respectively. The selected PAM sequences [(T)TTN on the sense strand or NAA (A) on the antisense strand] and the corresponding gRNA binding sequences are presented in red and green boxes. Right and left arrows signify the sense and complementary sequence, respectively, that are used. **(C)** Schematic illustration of MCMD workflow. MCMD employs four closely connected steps: rapid DNA extraction (15 min, STEP 1), GO-assisted multiplex RPA reaction (30 min, STEP 2), CRISPR–Cas12a-based trans-cleavage assay (8–10 min, STEP 3) and result readout (immediately in fluorescence and 3 min in LFB, STEP 4), which can be completed within 1 h.

Two sets of RPA primers targeting IS*6110* and IS*1081* were designed with Primer Premier 5.0 (Table 1). A BLAST analysis of the GenBank nucleotide database was performed to confirm the specificity of the primers. The gRNAs (Table 1; Figure 1B) were designed against the 20 nt sequence following the protospacer-adjacent motif (PAM) sequences [5’-(T) TTN-3′ on the sense strand or 5′-NAA(A)-3′ on the antisense strand] that served as Cas12a recognition sites. We used two ssDNAs (a fluorescent reporter and a biotin-labeled reporter) (Table 1).

**TABLE 1:** Sequences of primers, gRNAs and ssDNAs used in this study.

Primers/gRNAs/ssDNAs	Sequences (5′-3′)	Length
*Primers*		
IS*6110*-RPA-F^a^	ATC​AGT​GAG​GTC​GCC​CGT​CTA​CTT​GGT​GTT	30 nt^b^
IS*6110*-RPA-R^c^	CTT​CAG​CTC​AGC​GGA​TTC​TTC​GGT​CGT​G	28 nt
IS*1081*-RPA-F	CGC​CAG​GGC​AGC​TAT​TTC​CCG​GAC​TGG​CTG	30 nt
IS*1081*-RPA-R	CTT​GGA​AAG​CTT​TGT​CAC​ACC​AAG​TGT​TTC​GAC	33 nt
*GRNAs*		
IS*6110*-gRNA	UAAUUUCUACUAAGUGUAGAUGCUGCGCGGAGACGGUGCGU ^d^	41 nt
IS*1081*-gRNA	UAA​UUU​CUA​CUA​AGU​GUA​GAUGAC​CAG​GCG​CUC​CAU​CCG​GC	41 nt
*SsDNAs*		
Fluorescent reporter	5′-FAM-TATTATTATTATTT-BHQ1-3′	14 nt
Biotin-labeled reporter	5′-Biotin-TATTATTATTATTT-FAM-3′	14 nt

^a^ F, forward.

^b^ nt, nucleotide.

^c^ R, reverse.

^d^ Underlined sequence refers to the region complementary to the IS*6110* or IS*1081* target sequence.

### 2.3 Bacterial strains and genomic DNA extraction

The MTBC reference strain H37Rv was utilized during the establishment and clinical application of the MCMD technique. *Mycobacterium abscessus* was used as a negative control (NC). A total of 27 bacterial strains were used to determine the analytical specificity of MCMD (see Section 2.8). Genomic DNA was extracted using the QIAamp DNA Mini Kit (bacteria strains) or glass bead-based kit (sputum specimens) according to the manufacturer’s instructions and stored at −80°C before use. DNA quantity and purity were determined using ultraviolet spectrophotometry at 260 and 280 nm.

### 2.4 GO-assisted singlex and multiplex RPA assay

The Singlex RPA assay for IS*6110* (or IS*1081*) was performed according to the manual (TwistAmp Basic Kit). A 50 μL reaction mixture was prepared as follows: dry reaction pellet, 29.5 μL rehydration buffer, 0.48 µM forward and reverse primers, 1 ng μL^−1^ target template 2 μL (5 μL for sputum specimen), and 14 mM magnesium acetate. It has been reported that RPA is an error-prone reaction and usually yields non-specific amplicons and primer dimers (Munawar, 2022). Different concentrations of GO (0, 2, 4, 8, 16, 24, 32 μg mL^−1^) were added into the RPA mixtures at 38°C for 30 min to overcome the technical disadvantages of RPA. The reaction mixtures were incubated at 35.4°C–39.9°C (with 0.5°C–1.0°C intervals) for 20–40 min (with 10 min intervals) to determine the optimal reaction temperatures and amplification times for the RPA assay. Finally, the optimized operating conditions were used in a subsequent GO-assisted multiplex RPA assay, from which two sets of RPA primer (IS*6110* and IS*1081*) were added in one reaction system at a total concentration of 0.48 µM (1:1). *Mycobacterium abscessus* and double-distilled water (DDW) were used as NC and blank control (BC), respectively. Agarose gel electrophoresis (2.5%) was used to confirm the amplification of the RPA assay.

### 2.5 CRISPR–Cas12a-based trans-cleavage assay

The CRISPR–Cas12a-based trans-cleavage assay was adopted from previous studies (Chen et al., 2018; Li et al., 2018; Broughton et al., 2020) with some adjustments. Briefly, 41.7 nM of IS*6110*-gRNA and IS*1081*-gRNA (1:1) were preincubated with 33.3 nM LbCas12a in 1× NEBuffer 2.1 (NEB, MA, United States) at 37°C for 10 min to induce Cas12a-gRNA complexes that can be used immediately or stored at 4°C for up to 24 h. Subsequently, the trans-cleavage assay was conducted in a volume of 50 μL mixture, containing 25 μL 2 × NEBuffer 2.1, 13.5 μL Cas12a-gRNA complexes, 250 nM fluorescent reporter molecule, and 2 μL of the RPA products. This trans-cleavage assay was performed at 37°C, and the fluorescence signal was monitored for 20 min. The fluorescence signal increasing more than two-fold compared to BC at 10 min of the trans-cleavage assay was considered positive. A biotin-labeled reporter molecule was used instead of the fluorescent reporter for the LFB readout. After 8 min incubation at 37°C, the LFB assay was performed (see Section 2.6).

### 2.6 LFB assay

**FIGURE 2:**
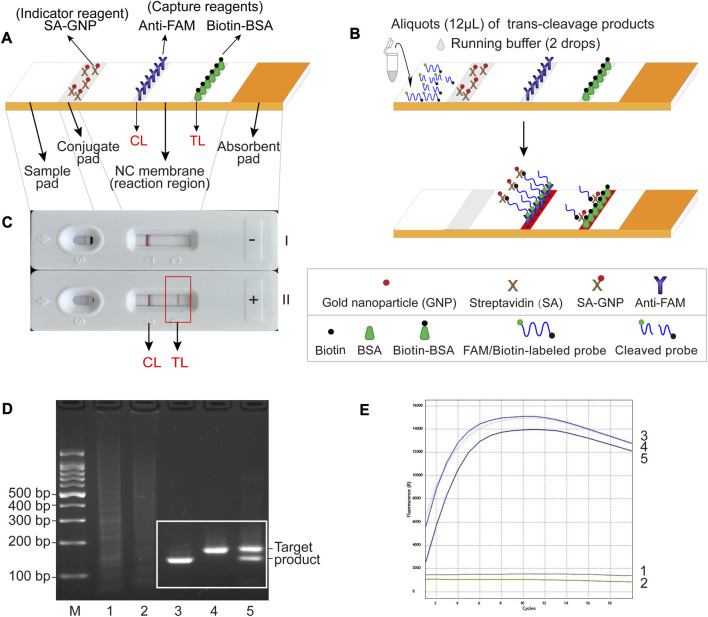
The schematic illustration of LFB assay used for visualization and feasibility validation of MCSD and MCMD. **(A)** Details of the LFB. The positions of indicator reagent (SA-GNP) and capture reagents (anti-FAM and biotin-BSA) of LFB are shown on the upper part of the diagram, and four main components (sample pad, conjugate pad, NC membrane and absorbent pad) and two lines (CL and TL) are labeled on the lower part of the diagram. **(B)** The schematic illustration of the LFB assay used for visualization. Twelve microliters of trans-cleavage products and two drops of running buffer were deposited onto the sample pad, and the results were visualized (develop red color) on CL and TL. **(C)** Interpretation of the LFB results. I, negative results (only CL appears); II, positive results (both CL and TL appear). **(D)** Agarose gel electrophoresis results of GO-assisted singlex and multiplex RPA assay. M, DNA marker; 1, blank control; 2, negative control; 3, GO-assisted IS*6110* RPA assay; 4; GO-assisted IS*1081* RPA assay; 5, GO-assisted multiplex RPA assay. **(E)** Real-time fluorescence images of MCSD and MCMD. 1, blank control; 2, negative control; 3, MCSD-IS*6110*; 4, MCSD-IS*1081*; 5, MCMD.

The LFB used for visual readout consists of several main components: sample pad, conjugate pad, nitrocellulose (NC) membrane (reaction region), and absorbent pad mounted on a backing card (Figure 2A). The streptavidin-gold nanoparticles (SA-GNPs) are adhered onto the conjugate pad and used as the indicator reagent, rabbit anti-fluorescein amide (anti-FAM) antibody and biotin-bovine serum albumin (biotin-BSA) are fixed onto the NC membrane and used as control line (CL) and test line (TL) capture reagents, respectively. Twelve microliters of trans-cleavage products and two drops of running buffer (100 mM phosphate-buffered solution, pH 7.4 with 1% Tween 20) were added to the sample pad. The detection results were visualized within 3 min as red bands on the NC membrane.

### 2.7 Analytical sensitivity of MTBC CRISPR–Cas12a-based Singlex detection (MCSD) and MCMD

The limit of detection (LoD) was determined using 10-fold serial dilutions of genomic DNA from reference strain H37Rv, ranging from 40,000 to 0.4 copies μL^−1^ to assess the analytical sensitivity of CRISPR–Cas12a-based singlex and multiplex detection for MTBC identification. DNA copy numbers per microliter were calculated using the following formula: (6.02 × 10^23^) × (ng μL^−1^ ×10^−9^)/(DNA length × 660). A volume of 2 µL from each DNA dilution was added to the RPA reaction mixture. Each dilution series was performed in triplicate. The lowest positive dilution (twice the fluorescence value of BC) in all replicates was considered the LoD.

### 2.8 Analytical specificity of MCMD

Reactions were conducted with genomic templates extracted from different bacterial strains, including 1 MTBC reference strain H37Rv, 1 *Mycobacterium bovis* Bacilli Calmette-Guerin (BCG), 8 clinical MTBC strains isolated from TB patients, 10 non-tuberculous mycobacteria (NTM) strains, and 7 non-mycobacteria strains to determine the analytical specificity of MCMD (Supplementary Table S1). Each strain was tested at least twice.

### 2.9 Application of MCMD in clinical specimens

This study was approved by the Ethical Committee of Beijing Children’s Hospital, Capital Medical University (2020-k-163). A total of 107 patients with suspected active pulmonary TB from Beijing Chest Hospital were enrolled in this study from May to June 2022. The need for informed consent was waived because the sputum specimens used in this study were leftover samples from clinical microbiology tests. According to the Chinese National Standard on Diagnosis for Pulmonary Tuberculosis (WS288-2017, National Health and Family Planning Commission of the People’s Republic of China, 2017), the patients were categorized according to the composite reference standard (CRS), which combines the clinical and laboratory diagnostic criteria: (a) definite TB/laboratory-confirmed TB, patients with bacteriological confirmation of MTB (culture, smear, nucleic acid detection, or histopathological evidence positive); (b) probable TB/clinical diagnosed TB, patients with radiologic findings suggestive of TB plus at least one of the following: TB clinical symptoms or signs, positive tuberculin skin test (TST) or IGRA, bronchoscopy or histopathology consistent with TB; and (c) non-TB, patients diagnosed as other diseases and improved in the absence of anti-TB treatment.

Sputum samples (2–3 mL) collected from patients were utilized for TB culture, Xpert, and MCMD assays. A tuberculosis culture was performed using Bactec MGIT 960 system (Becton Dickinson, MD, United States). Xpert (Cepheid, CA, United States) was performed following the manufacturer’s protocol. For MCMD, sputum samples were decontaminated and liquefied by adding an equal volume of 4% NaOH, and sputum DNA was extracted using the glass bead-based kit. Subsequently, 5 µL DNA solution was added to the RPA reaction mixture. All samples were detected in duplicate in multiple independent batches, and each batch included a positive control (PC) (H37Rv DNA as the template) and BC. The results from MCMD were compared to TB culture and Xpert to evaluate the diagnostic performance of this new technique.

## 3 Results

### 3.1 Schematic mechanism of MCMD

The MCMD integrated GO-assisted multiplex RPA assay that simultaneously targets IS*6110* and IS*1081* with a CRISPR–Cas12a-based trans-cleavage assay at fixed temperatures for MTBC nucleic acid detection. First, the extracted MTBC DNA templates are pre-amplified with three proteases [recombinase, single strand DNA binding protein (SSB), and DNA polymerase] at 37–42°C within 30 min (Figure 1A). Next, in the trans-cleavage stage, the PAM site in the RPA amplicon can guide the CRISPR–Cas12a-IS*6110*(or IS*1081*)-gRNA complex to its location (Figures 1B, C), activating the CRISPR–Cas12a effector. The activated Cas12a has trans-cleavage activity against ssDNA reporter. Finally, the digestion of reporter molecules can be detected via fluorescence or LFB, which confirms the presence of the target genome MTBC. The entire MCMD assay, including rapid DNA extraction (15 min, STEP 1), GO-assisted multiplex RPA reaction (30 min, STEP 2), CRISPR–Cas12a-based trans-cleavage assay (8–10 min, STEP 3), and result readout (immediately in fluorescence and 3 min in LFB, STEP 4), can be completed within 60 min.

For fluorescence readout, the fluorescent reporter, labeled at the 5′-end with a FAM fluorophore (6-carboxyfluorescein) and at the 3′-end with a black hole quencher (BHQ1), is cleaved by the activated Cas12a and released from its quencher, increasing fluorescent signaling (Figure 1C STEP 3, STEP 4). Thus, CRISPR–Cas12a-based trans-cleavage and fluorescence readout can be performed simultaneously.

### 3.2 Schematic illustration of LFB assay for visualization

For the LFB readout, when the cleaved products are added to the sample pad followed by the running buffer, the running buffer travels along the biosensor through capillary action, rehydrating the indicator reagent (SA-GNPs) in the conjugate pad (Figure 2B). In the negative sample, the uncleaved biotin-labeled reporter molecule (5′-Biotin-TATTATTATTATTT-FAM-3′) binds SA-GNP (via biotin at the 5′ end of the reporter), and it is captured by anti-FAM immobilized on the CL (via FAM at the 3′ end of the reporter), then CL is displayed in red for visualization. In the positive sample, the biotin-labeled reporter cleaved by activated Cas12a binds SA-GNP. It is then captured by biotin-BSA immobilized on the TL, and then TL is visualized. Therefore, the principle of visualization in the LFB assay is that the biotin–SA-GNPs complexes seized by capture reagents (anti-FAM on CL or biotin-BSA on TL) develop red bands (Figure 2C).

### 3.3 Establishment and optimization of RPA assay

According to our previous study, different concentrations of GO ranging from 4 μg mL^−1^–32 μg mL^−1^ were added to the GO-assisted singlex RPA assays to improve the specificity of RPA (Wang Y et al., 2020). As shown in Supplementary Figure S1A and S1B, GO concentrations ranging from 8 μg mL^−1^–16 μg mL^−1^ exhibited good specificity performance with no obvious decrease in RPA product yield. Concentrations less than 4 μg mL^−1^ showed no improvement in amplification specificity, while concentrations above 24 μg mL^−1^ partially inhibited the amplification reactions. Therefore, we recommend 8 μg mL^−1^ as the optimal concentration for GO-assisted RPA assays.

According to Supplementary Figures S1C, S1D, the optimal reaction temperatures for IS*6110*-RPA and IS*1081*-RPA primers were 37.4°C–39.4°C and 37.9°C–38.9°C, respectively. Thus, an ideal temperature range of 37.9°C–38.9°C was recommended for the subsequent GO-assisted multiplex RPA assays, using both IS*6110* and IS*1081* RPA primers in one reaction. As can be seen form Supplementary Figure S1E, RPA products increased with the duration of amplification. Considering both a shorter test time and a higher amplification yield, the optimal amplification time was recommended to be 30 min. Figure 2D shows that specific amplification products were slightly reduced in the GO-assisted multiplex RPA assay compared to the two GO-assisted singlex RPA assays. This occurrence is presumed to be due to interference among the primers, particularly when multiple pairs of RPA primers are present. Whether this could lead to an analytical loss of sensitivity requires further experiments (see Section 3.5).

### 3.4 Trans-cleavage assay of MCSD and MCMD

MCMD was performed in parallel with two singlex detections (MCSD-IS*6110* and MCSD-IS*1081*). The high fluorescent signals in Figure 2E indicated that these assays could identify MTBC by targeting IS*6110* (or IS*1081*) or both. Fluorescence signals from MTBC CRISPR–Cas12a detections on various RPA products were detectable in 1 min, increased rapidly within 8 min, and decreased slowly after 12 min (Figure 2E). Finally, an optimized reaction time of 8–12 min was recommended for the trans-cleavage stage.

### 3.5 Analytical sensitivity of MCSD and MCMD

**FIGURE 3:**
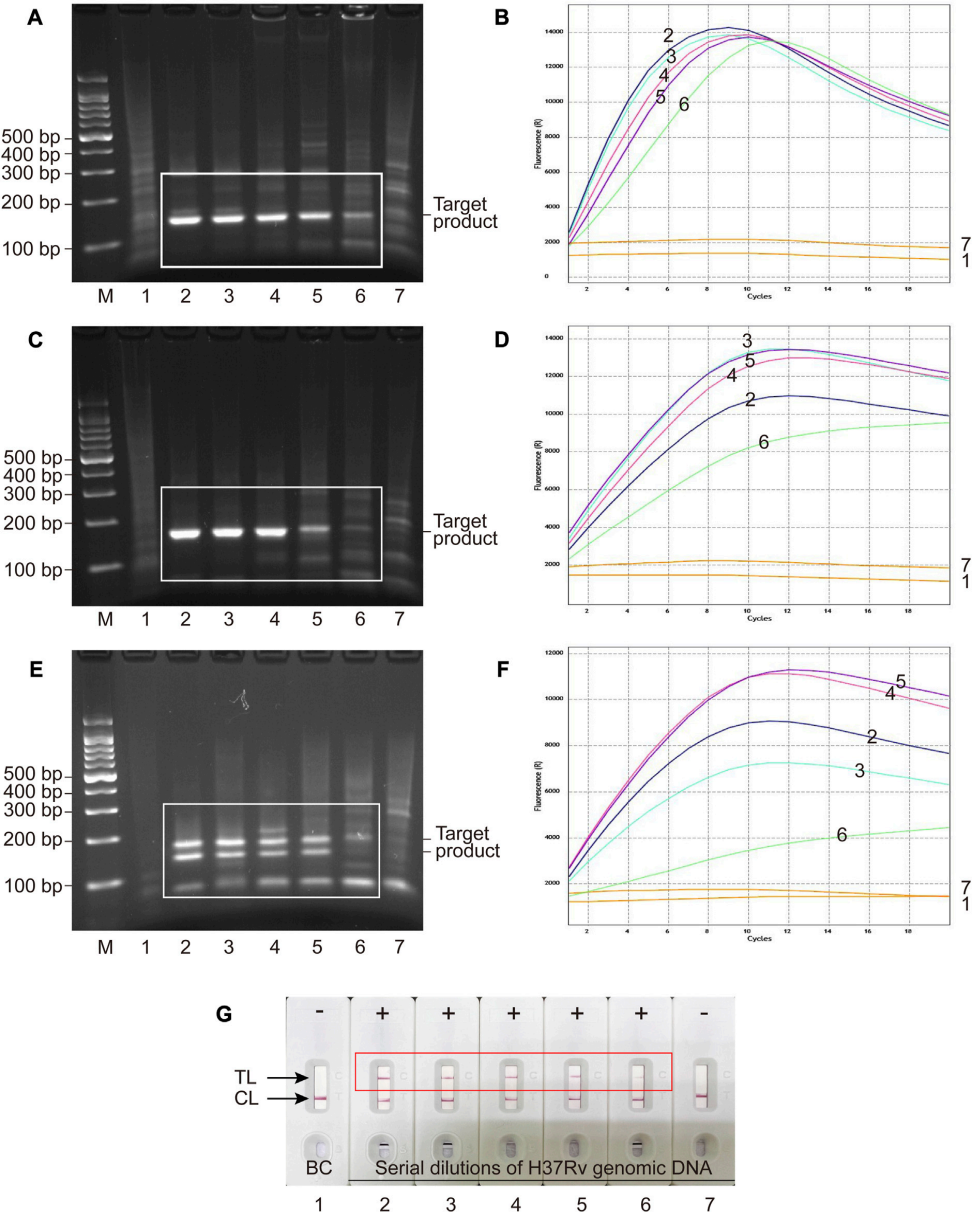
Analytical sensitivity of MCSD and MCMD as determined by serially diluted genomic DNA of H37Rv. **(A,C,E)** Agarose gel electrophoresis of GO-assisted IS*6110* RPA assay, IS*1081* RPA assay and multiplex RPA assay. **(B,D,F)** Real-time fluorescence images of MCSD-IS*6110*, MCSD-IS*1081* and MCMD. **(G)** LFB applied for reporting the results of MCMD. M, DNA marker; 1, blank control; 2 to 7, serial dilutions (40,000 copies μL^−1^, 4,000 copies μL^−1^, 400 copies μL^−1^, 40 copies μL^−1^, 4 copies μL^−1^, 0.4 copy μL^−1^) of H37Rv genomic DNA.

Serial dilutions of H37Rv genomic DNA (40,000 to 0.4 copies μL^−1^) were used for analytical sensitivity determination of MCSD and MCMD. Electrophoresis and fluorescence results showed that two singlex detections (MCSD) and the multiplex detection (MCMD) were all sensitive with the LoDs of 4 copies μL^−1^ (Figures 3A–F). Figure 3G showed that the MCMD LFB assay also had a similar LoD value. Furthermore, the electrophoretic band brightness shown in Figures 3A, C, E indicates a dependency on the target amount in the RPA assay. However, such typical trend was not observed in the fluorescence intensity shown in Figures 3D, F. Two reasons may explain this result. First, this may be attributed to the differences in the targets of RPA assay and fluorescence detection. The RPA assay targeted serial dilutions of H37Rv DNA, whereas the fluorescence detection targeted the RPA products, which were not serial dilutions of DNA. Second, the cycle threshold (Ct) value (rather than the fluorescence intensity) is an inverse measure of the template load. The trans-cleavage reactions initiate almost instantly after the mixing of the reaction components at room temperature, resulting in the inability to read Ct values of RPA products in the resulting figures. While the curve height in Figures 3B, D, F represents the fluorescence intensity, it does not directly correspond to the concentrations of the templates. Altogether, MCMD that can juggle multiplex and unimpaired sensitivity was recommended for the subsequent experiments, including analytical specificity testing and clinical application.

### 3.6 Analytical specificity of MCMD

Genomic DNA extracted from 27 bacterial strains shown in Supplementary Table S1 was used to validate the analytical specificity of MCMD. As shown in Supplementary Figure S2, MTBC strains, including H37Rv strain (as a PC), BCG strain, and clinical strains from TB patients, gave positive results. In contrast, NTM strains, non-mycobacteria strains, and DDW (as a BC) showed negative results, demonstrating the absence of cross-reactions. Only MTBC genomic DNA could be detected using MCMD, indicating the extremely high specificity (100%) of this method.

### 3.7 Application of MCMD in clinical samples

A total of 107 pulmonary TB patients and 40 non-TB patients were included in this study. The proportion of males in the TB group was 64.5% (69/107), and the mean age was 50.84 years. The TB group comprised 79 laboratory-confirmed and 28 clinically diagnosed TB patients. In the non-TB group, 57.5% (23/40) were males, and the average age was 68.30. The diagnostic specificity of MCMD was evaluated in these non-TB patients, which consisted of 30 patients with bacterial pneumonia, seven with non-infectious inflammatory diseases, and three with malignancies. All samples were sputum specimens.

As shown in Table 2, 107 TB patients were classified into two groups, the culture-positive group (67/107) and the culture-negative group (40/107). For the culture-positive samples, the diagnostic sensitivity of Xpert and MCMD was 85.1% (57/67) and 95.5% (64/67), respectively. For the culture-negative samples, the diagnostic sensitivity of Xpert and MCMD was 27.5% (11/40) and 40.0% (16/40), respectively. Among all TB patients in this study, the pooled diagnostic sensitivity of culture, Xpert, and MCMD was 62.6% (67/107), 63.6% (68/107), and 74.8% (80/107), respectively. These results indicate that MCMD exhibits higher sensitivity compared to culture and Xpert, making it a more promising diagnostic tool for TB. In addition, the diagnostic specificity was 100% (40/40) for Xpert and MCMD when testing samples from non-TB patients.

**TABLE 2:** Comparison of diagnostic performance of different methods for MTBC detection in TB patients.

	Diagnostic sensitivity, n^a^ (%), (pulmonary TB patients)			Diagnostic specificity, n (%), (non-TB patients) (N = 40)
	Culture positive (N = 67)	Culture negative (N = 40)	Total (N = 107)	
Xpert	57 (85.1)	11 (27.5)	68 (63.6)	40 (100)
MCMD	64 (95.5)	16 (40.0)	80 (74.8)	40 (100)

^a^ n, number of samples with positive results using a specific method.

## 4 Discussion

The CRISPR–Cas12a-based multiplex detection targeting two conserved sequences (IS*6110* and IS*1081*) was performed at constant temperatures (∼37°C) and could be completed within 1 h. The LoD of this technique reached single-digit copies μL^−1^ (4 copies μL^−1^), and no cross-reaction with non-MTBC bacteria strains was observed. The MCMD showed a diagnostic sensitivity of 74.8% and specificity of 100% in clinical samples compared to Xpert (63.6%, 100%).

The present study had several strengths. First, we employed an improved RPA assay with GO in the preamplification phase and exhibited good specificity performance with reduced non-specific products. RPA usually generates multiple amplified bands for one target sequence, making it difficult to establish an efficient multiplex RPA assay with multiple primer pairs. Our study and other research groups reported that GO, a single-atom-thick sheet of 2D carbon nanomaterial, could reduce primer-dimers and non-specific fragment formation in multiple-round or multiplex amplification (Wang et al., 2017; Wang Y et al., 2020). Given the strong non-covalent binding of water-soluble GO and nucleobases, enzymes, and aromatic compounds, the GO mechanism involved in the RPA assay can be as follows (Pena-Bahamonde et al., 2018; Wang Y et al., 2020): first, the negatively charged GO can rapidly attract the RPA reaction components bearing positive charges (e.g., recombinase, SSB, DNA polymerase, and Mg^2+^) on the GO monolayer surface; second, the negatively charged RPA reaction components (e.g., nucleic acid templates, primers, and dNTPs) are attracted to the positively charged molecules on GO surface; then GO facilitates annealing of RPA primers to the target templates and extension. Consequently, the suppression of mismatched primer-template complex formation and the reduction of primer-dimers ultimately enhance the amplification specificity of RPA.

Notably, the unique strength of MCMD is its property of multiplex detection. The selection of a suitable target gene is of great significance for the high sensitivity and specificity of a diagnostic assay. So far, many conserved sequences, such as IS*6110*, IS*1081*, *MPB64*, *sdaA*, and *gyrB*, have been utilized as diagnostic targets for TB, of which IS*6110* is the most widely used (Yu et al., 2018; Acharya et al., 2020; Huang et al., 2021; Wang X et al., 2021). IS*6110* is a multi-copy insertion sequence among MTBC stains (e.g., 16 copies in H37Rv) that can increase sensitivity. However, low/no copies of IS*6110* are found in strains isolated from Southeast Asia, Europe, and America (Arora et al., 2020; Comin et al., 2021). Therefore, the techniques using a single target IS*6110* may be unsuitable in these areas. Our MCMD that combined another target IS*1081* (lower copy numbers, e.g., 5–6 copies in H37Rv) with IS*6110* can both avoid false negatives in strains with low/no copies of IS*6110* and as well as retain high sensitivity from IS*6110*. This is also why Xpert Ultra incorporated the two different multi-copy amplification targets (IS*6110* and IS*1081*) for improved detection of MTBC (Kay et al., 2022; Tang et al., 2023).

In comparison to PCR-based techniques, isothermal amplification methods eliminate the need for sophisticated equipment such as thermal cyclers and can significantly reduce amplification time (∼30 min). Moreover, Cas endonucleases with collateral trans-cleavage activity can amplify cleavage signals and exhibit ultra-high sensitivity when combined with a pre-amplification step. Additionally, multiplex techniques have the distinct advantage of enabling the detection of multiple targets within a single assay. This capability is crucial for achieving precise and reliable diagnosis, reducing costs, and minimizing sample volume requirements. However, to date, some studies have employed isothermal amplification and Cas12a-based trans-cleavage in nucleic acid detection of MTBC (Ai et al., 2019; Xu et al., 2020; Sam et al., 2021; Wang Y et al., 2021), but no study enabled multiplex detection in one assay except for this present study (Supplementary Table S2). In details, studies from Ai et al. (2019), Sam et al. (2021) and Wang Y et al. (2021) used IS*6110* as the only target, and the study from Xu et al. (2020) used IS*1081* as the only target. Compared to our study, Ai et al. (2019), Sam et al. (2021) and Xu et al. (2020) did not avoid the use of large detection equipment (e.g., real-time PCR instrument), and LAMP used by Sam et al. (2021) and Wang Y et al. (2021) requires higher temperature (60°C–68°C), longer reaction time (40–80 min), and more complex primers (six primers for one target gene). The only IS*1081*-targeted study by Xu et al. (2020) exhibited a lower sensitivity with at least 20-fold higher LoD than other IS*6110*-targeted studies (Ai et al., 2019; Sam et al., 2021; Wang Y et al., 2021; this study). Our MCMD, which juggled multiplex and high sensitivity (4 copies μL^−1^ LoD), had advantages of simplicity (only heating block and two pairs of primers), rapidity (1 h) and specificity (reduced non-specific amplicons by simply adding GO). It can even use body temperature for amplification and trans-cleavage and use naked eyes for readout. In comparison to the widely-used Xpert, our MCMD technique demonstrated higher diagnostic sensitivity with an increase of >10 percentage points, while maintaining similar diagnostic specificity. Moreover, the MCMD technique offers significant cost advantages, with costs of $10 compared to $80 for Xpert in China. Additionally, the MCMD technique requires smaller sample volumes of 200 μL, in contrast to the 1–6 mL volume required by Xpert. These findings underscore the great potential of this novel technique for POT testing of TB in resource-limited countries.

In recent years, biosensors based on the CRISPR–Cas system [e.g., SHERLOCK (Gootenberg et al., 2017), DETECTR (Chen et al., 2018), HOLMES (Li et al., 2018), Cas14-DETECTR (Harrington et al., 2018), HOLMESv2 (Li et al., 2019)] have shown excellent performance in POC testing. A comparison of different CRISPR-based biosensors is shown in Supplementary Table S3. Considering the complex guide RNA design and signaling mechanism, CRISPR–Cas9 is no longer the preferred option for CRISPR diagnostics (Pardee et al., 2016; Guk et al., 2017; Zhang et al., 2017; Huang et al., 2018; Qiu et al., 2018; Zhou et al., 2018; Hajian et al., 2019; Wang et al., 2019; Wang X et al., 2020; Zhang et al., 2022). Compared to Cas9, Cas12 and Cas13 have been widely used for CRISPR diagnostics due to their unique collateral cleavage activity, which greatly simplifies signal generation (Gootenberg et al., 2017; Chen et al., 2018; Gootenberg et al., 2018; Harrington et al., 2018; Li et al., 2018; Dai et al., 2019; Li et al., 2019; Joung et al., 2020; Liu et al., 2021). Cas12a and Cas12b directly target double-stranded DNA (dsDNA) and are useful for DNA sequence detection and genotyping. Cas12f (originally denoted Cas14) shows stronger specificity and enables SNP detection with high fidelity. The multiplexing potential of Cas13 allows for the detection of multiple analytes. Some Cas effectors not restricted by PAM [e.g., uncultured archaeon (Un1) Cas12f1 (initially named Cas14a1) (Harrington et al., 2018), Cas13a, Cas13b] result in more application space in target sequences without PAM. Researchers can reasonably select biosensor types according to their experimental requirements.

Although this study had several strengths, it also had some limitations. First, the sputum samples were all collected from a tertiary hospital specializing in tuberculosis. A multi-center prospective study should be conducted to provide high-level evidence on the diagnostic performance of MCMD. Second, only sputum samples, the most commonly used and most accessible specimen for adult TB patients, were evaluated in this study. Other paucibacillary samples (such as pleural effusion and cerebrospinal fluid) and specific populations (such as children and HIV-positive patients) should also be considered for future implementation. Third, it is recommended to design RPA primers with the incorporated PAM sequence in future studies. For CRISPR-Cas12 assays, it is essential that the target sequence contains a PAM site at the appropriate location to enable the activation of the Cas12-gRNA complex. Meanwhile, the MTBC genome is a high-GC genome, and the target gene may not have enough suitable PAM sites. Thus, improving primer design removes the limitation imposed by the PAM sequence and facilitates the screening of efficient gRNAs. Finally, in the current investigation, single-signal output biosensors (including LFB and fluorescent biosensor) were employed to indicate the presence of MTBC in the sample. However, in future studies, the utilization of distinct fluorescence or double-labeled LFB may enable differentiation between IS*6110* and IS*1081*, thereby providing additional strain-specific information. Despite major advances in the applications of CRISPR-based biosensors, many challenges remain in this promising area. Newer Cas enzymes, more robust readout systems, and more flexible strategies of signal conversion and amplification will represent the possible directions of improvement in future.

In this study, we have achieved the simultaneous detection of multiple targets from MTBC using a CRISPR-Cas-based detection method. This MCMD first combined an improved GO-assisted multiplex RPA targeting IS*6110* and IS*1081* with the CRISPR–Cas12a-based trans-cleavage assay identified by a fluorescent biosensor or LFB. Due to its multiplex property and freedom from large equipment, this MCMD is ideal for detecting strains with low/no copies of IS*6110* and MTBC POC testing in resource-limited countries.

## Data Availability

The raw data supporting the conclusion of this article will be made available by the authors, without undue reservation.
