# Supplementary figures and images for: Development and preliminary assessment of a CRISPR–Cas12a-based multiplex detection of Mycobacterium tuberculosis complex
Source: Front Bioeng Biotechnol. 2023 Aug 25;11:1233353. doi: 10.3389/fbioe.2023.1233353 (PMC10497956; doi:10.3389/fbioe.2023.1233353)

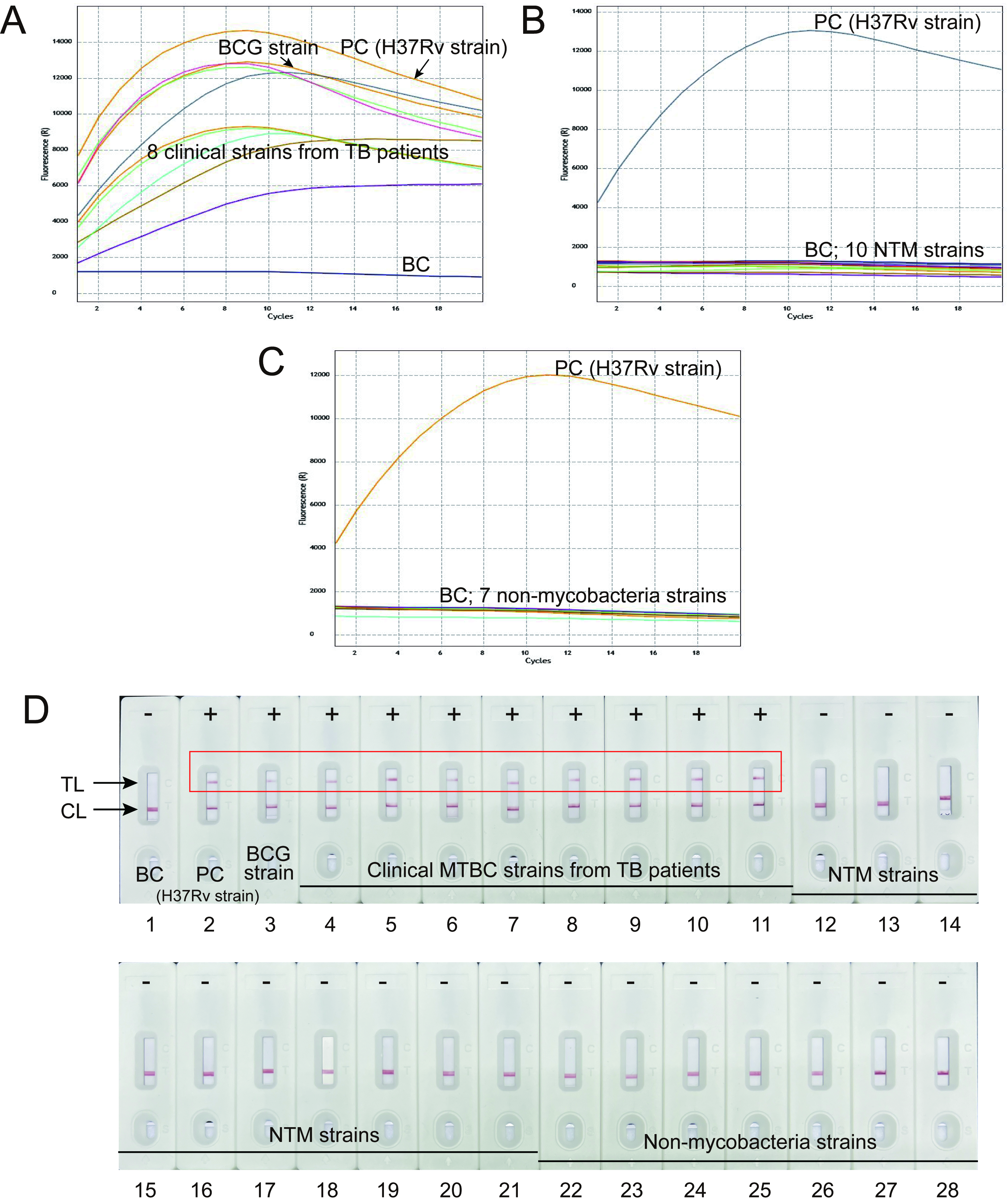

Supplement: Supplementary file 1 [file Image2.TIF]

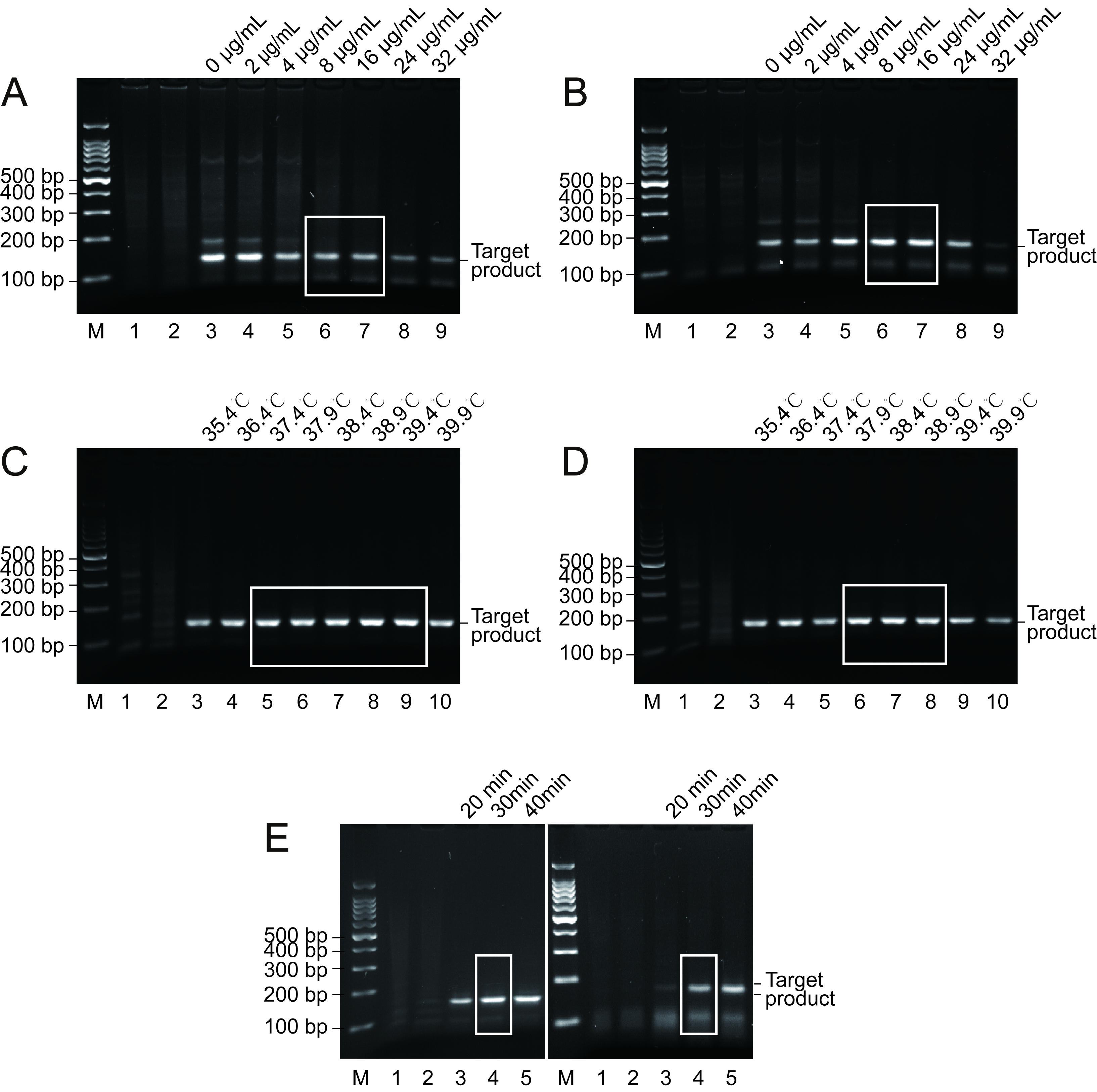

Supplement: Supplementary file 2 [file Image1.TIF]
