# Supplementary material for: Development and preliminary assessment of a CRISPR–Cas12a-based multiplex detection of Mycobacterium tuberculosis complex
Source: Front Bioeng Biotechnol. 2023 Aug 25;11:1233353. doi: 10.3389/fbioe.2023.1233353 (PMC10497956; doi:10.3389/fbioe.2023.1233353)
Supplement: Supplementary file 3 [file DataSheet1.docx]

**Supplementary Table**

TABLE S1. Bacteria strains used in this study and the analytical specificity of MCMD.

| Bacteria | Strain (source) | No. of strains | MCMD |
| --- | --- | --- | --- |
| *M. tuberculosis* | H37Rv (BCH-NTCL^a^) | 1 | P^b^ |
| *M. bovis BCG* | BCG (BCH-NTCL) | 1 | P |
| *M. tuberculosis* | Isolated strains (BCH-NTCL) | 8 | P |
| *M. chelonei* | Isolated strain (BCH-NTCL) | 1 | N^c^ |
| *M. avium* | Isolated strain (BCH-NTCL) | 1 | N |
| *M.gordonae* | Isolated strain (BCH-NTCL) | 1 | N |
| *M. abscessus* | Isolated strain (BCH-NTCL) | 1 | N |
| *M. kansasii* | Isolated strain (BCH-NTCL) | 1 | N |
| *M. fortuitum* | Isolated strain (BCH-NTCL) | 1 | N |
| *M. intracellulare* | Isolated strain (BCH-NTCL) | 1 | N |
| *M. marcellus* | Isolated strain (BCH-NTCL) | 1 | N |
| *M. malmoense* | Isolated strain (BCH-NTCL) | 1 | N |
| *M. porcinum* | Isolated strain (BCH-NTCL) | 1 | N |
| *Klebsiella pneumoniae* | Isolated strains (BCH^d^) | 1 | N |
| *Streptococcus pneumoniae* | Isolated strains (BCH) | 1 | N |
| *Mycoplasma pneumoniae* | Isolated strains (BCH) | 1 | N |
| *Haemophilus influenzae* | Isolated strains (BCH) | 1 | N |
| *Escherichia coli* | Isolated strains (BCH) | 1 | N |
| *Staphylococcus aureus* | Isolated strains (BCH) | 1 | N |
| *Group B streptococcus* | Isolated strains (BCH) | 1 | N |

Reference strain H37Rv and DDW were used as PC and BC. Each strain was tested in duplicate.

^a^BCH-NTCL, National Tuberculosis Clinical Laboratory, Beijing Chest Hospital; ^b^P, positive; ^c^N, negative; ^d^BCH, Beijing Children’s Hospital.

TABLE S2. Use of isothermal amplification methods plus CRISPR technique for the rapid detection of MTBC.

| References | Targets | Method principle | Instruments | Time to result | Readout methods | LOD^a^ | Technical strengths | Technical weaknesses |
| --- | --- | --- | --- | --- | --- | --- | --- | --- |
| *Isothermal amplification methods plus CRISPR technique* | | | | | | | | |
| Ai et al., 2019 | IS*6110* | RPACRISPR–Cas12a cleavage real-time fluorescence | Heating block;  real-time PCR instrument | 90 min (RPA 30 min; CRISPR 20 min) | Instrument readout | 5 copies μL^-1^ | 1. Simple primer design 2. One pair of primers for one target | 1. Singlex detection 2. PAM dependence 3. Special instrument requirements |
| Sam et al., 2021 | IS*6110* | LAMPCRISPR–Cas12b cleavage real-time fluorescence | Heating block;  real-time PCR instrument | 120 min (LAMP 80 min; CRISPR 10 min) | Instrument readout | 1.3 copies μL^-1^ | Lower LOD (at the expense of a longer amplification time) | 1. Singlex detection 2. PAM dependence 3. Complex primer design 4. Numerous primers (one set of 6 primers for one target) 5. Special instrument requirements 6. Long amplification time |
| Wang et al., 2021 | IS*6110* | Modified LAMP CRISPR–Cas12a cleavage real-time fluorescence or lateral flow | Heating block;  real-time PCR instrument or no detection instrument | 60 min (LAMP 40 min; CRISPR 5 min) | Instrument or Visual readout | 1. fg reaction^-1^ | 1. PAM independence by an engineered FIP primer 2. Modest instrument requirements | 1. Singlex detection 2. Complex primer design 3. Numerous primers (one set of 6 primers for one target) |
| Xu et al., 2020 | IS*1081* | RPACRISPR–Cas12a cleavage real-time fluorescence | Heating block;  real-time PCR instrument | 240 min (RPA 60 min; CRISPR 120 min) | Instrument readout | 4.48 fmol L^-1^ | 1. Simple primer design 2. One pair of primers for one target | 1. Singlex detection 2. PAM dependence 3. Special instrument requirements 4. Long turnaround time 5. Higher LOD |
| *Multiplex isothermal amplification methods plus CRISPR technique* | | | | | | | | |
| This study | IS*6110*+ IS*1081* | GO-assisted multiplex RPA CRISPR–Cas12a cleavage real-time fluorescence or lateral flow | Heating block;  real-time PCR instrument or no detection instrument | 60 min (RPA 30 min; CRISPR 10 min) | Instrument or Visual readout | 4 copies μL^-1^ | 1. Multiplex detection 2. Simple primer design 3. Two pairs of primers for two targets 4. Fewer non-specific amplicons by simply adding GO 5. Modest instrument requirements or even instrument-free | PAM dependence |

^a^The description of LOD presented in the original article is adopted. The LODs of various detection methods may not necessarily be comparable, because some studies do not clarify how their data were obtained. Generally, LOD is influenced by sample preparation method, amplification method, incubation time, and readouts.

TABLE S3. Comparison of different CRISPR-based biosensors for POC testing.

| CRISPR-based biosensors | Cas effectors | tracrRNA^a^ | PAM/PFS^b^ | Nuclease domains | Cleavage activity | Targets | Enzymatic characteristics | Applicable scope | System name (Refs) |
| --- | --- | --- | --- | --- | --- | --- | --- | --- | --- |
| CRISPR–Cas9 (/Cas9n^c^/ dCas9^d^)-based biosensors | Cas9 | Required | 3’G-rich PAM | HNH, RuvC | cis-cleavage | dsDNA (DSBs^e^; blunt ends) | 1. PAM-dependent dsDNA targeting/cleavage activity and no collateral cleavage activity 2. Complex guide RNA design and signaling mechanism (integration with other techniques to indicate detection readout) 3. A nickase version (i.e., Cas9n) available (not in Cas12/Cas13) | *No longer the preferred option for CRISPR diagnostics*   1. Pathogens detection 2. SNP^g^ detection | NASBACC (Pardee et al., 2016), CAS-EXPAR (Huang et al., 2018) |
|  | Cas9n |  |  | HNH, RuvC containing one mutation | cis-cleavage | dsDNA (SSBs^f^) |  |  | CRISDA (Zhou et al., 2018), Cas9nAR (Wang et al., 2019) |
|  | dCas9 |  |  | HNH, RuvC containing two mutations | None | dsDNA (No DNA breaks) |  |  | PC reporter (Zhang et al., 2017; Zhang et al., 2022 ^h^), dCas9/sgRNA-SG I based DNA-FISH (Guk et al., 2017), RCA-CRISPR-split-HRP (RCH) (Qiu et al., 2018), CRISPR-Chip (Hajian et al., 2019), CASLFA (Wang et al., 2020) |
| CRISPR–Cas12-based biosensors | e.g., Cas12a | Not required | 5’T-rich PAM (targeting dsDNA); PAM-independence (targeting ssDNA) | RuvC | cis-cleavage; trans-cleavage | dsDNA (DSBs; staggered ends); ssDNA | 1. PAM-dependent dsDNA targeting/cleavage activity and collateral cleavage activity against ssDNA 2. More heat resistant Cas12b (vs. Cas12a) | *Widely used for CRISPR diagnostics*   1. Especially suitable for DNA sequence detection and genotyping 2. SNP detection 3. Cancer screening 4. One-pot detection system | DETECTR (Chen et al., 2018), HOLMES (Li et al., 2018), E-CRISPR (Dai et al., 2019) |
|  | e.g., Cas12b | Required |  |  |  |  |  |  | HOLMESv2 (Li et al., 2019), STOP (Joung et al., 2020) |
|  | e.g., Cas12f (originally denoted Cas14) | Required | 5’T or C-rich PAM (targeting dsDNA); PAM-independence (targeting ssDNA) | RuvC | cis-cleavage; trans-cleavage | dsDNA (partly effectors; DSBs; staggered ends); ssDNA | 1. PAM-independent ssDNA targeting and collateral cleavage activity 2. Stronger specificity (vs. Cas12a/Cas12b) 3. Smaller size (vs. other Cas effectors) | 1. Especially suitable for ssDNA pathogens detection 2. High-fidelity SNP detection | Cas14-DETECTR (Harrington et al., 2018) |
| Cas13-based biosensors | e.g., Cas13a, Cas13b | Not required | 3’non G PFS | 2×HEPN | cis-cleavage; trans-cleavage | ssRNA | 1. Require DNA transcription into RNA 2. PAM-independent ssRNA targeting and collateral cleavage activity | 1. Pathogens detection and genotyping 2. Cancer screening 3. Multiple analyte detection 4. One-pot detection system | SHERLOCK (Gootenberg et al., 2017), SHERLOCKv2 (Gootenberg et al., 2018), FIND-IT (Liu et al., 2021) |

^a^tracrRNA, trans-activating CRISPR RNA; ^b^PFS, Protospacer flanking sequence; ^c^Cas9n, Cas9 nickase; ^d^dCas9, dead Cas9; ^e^DSBs, double-stranded breaks; ^f^SSBs, single-stranded breaks; ^g^SNP, single nucleotide polymorphism.

^h^applied to the detection of MTB.

**Supplementary Figure**

**Figure Captions**

FIGURE S1.

Optimization of RPA assay. **(A)** The effect of GO on IS*6110* RPA assay (38.0℃). **(B)** The effect of GO on IS*1081* RPA assay (38.0℃). M, DNA marker; 1, blank control; 2, negative control; 3 to 9, different GO concentrations (from 0 to 32 μg mL^-1^). **(C)** Optimal reaction temperature for IS*6110*-RPA primers (GO=8 μg mL^-1^). **(D)** Optimal reaction temperature for IS*1081*-RPA primers (GO=8 μg mL^-1^). M, DNA marker; 1, blank control; 2, negative control; 3 to 10, different reaction temperatures (35.4~39.9 ℃). **(E)** Optimal amplification time for IS*6110* (left panel) and IS*1081* (right panel) RPA assay (GO=8 μg mL^-1^, 38.0℃).

FIGURE S2.

Analytical specificity of MCMD as determined by genomic DNA extracted from 27 bacterial strains. **(A)** Real-time fluorescence images of MTBC strains. **(B)** Real-time fluorescence images of NTM strains. **(C)** Real-time fluorescence images of non-mycobacteria strains. **(D)** LFB results of bacteria strains used in this study. BC, blank control; PC, positive control.
